# Phosphatidylcholine Liposomes Down-Modulate CD4 Expression Reducing HIV Entry in Human Type-1 Macrophages

**DOI:** 10.3389/fimmu.2022.830788

**Published:** 2022-05-19

**Authors:** Federica De Santis, Ana Borrajo Lopez, Sara Virtuoso, Noemi Poerio, Patrizia Saccomandi, Tommaso Olimpieri, Leonardo Duca, Lucia Henrici De Angelis, Katia Aquilano, Marco Maria D’Andrea, Stefano Aquaro, Alessandra Borsetti, Francesca Ceccherini-Silberstein, Maurizio Fraziano

**Affiliations:** ^1^ Dipartimento di Biologia, Università degli Studi di Roma “Tor Vergata”, Roma, Italy; ^2^ Dipartimento di Medicina Sperimentale, Università degli Studi di Roma “Tor Vergata”, Roma, Italy; ^3^ Centro Nazionale per la ricerca su HIV/AIDS, Istituto Superiore di Sanità, Roma, Italy; ^4^ Dipartimento di Biotecnologie Mediche, Università di Siena, Siena, Italy; ^5^ Dipartimento di Farmacia e Scienze della Salute e della Nutrizione, Università della Calabria, Rende, Italy

**Keywords:** host-directed therapy, liposome, phosphatidylcholine, macrophage, HIV entry

## Abstract

A strategy adopted to combat human immunodeficiency virus type-1 (HIV-1) infection is based on interfering with virus entry into target cells. In this study, we found that phosphatidylcholine (PC) liposomes reduced the expression of the CD4 receptor in human primary type-1 macrophages but not in CD4^+^ T cells. The down-regulation was specific to CD4, as any effect was not observed in CCR5 membrane expression. Moreover, the reduction of membrane CD4 expression required the Ca^2+^-independent protein kinase C (PKC), which in turn mediated serine phosphorylation in the intracytoplasmic tail of the CD4 receptor. Serine phosphorylation of CD4 was also associated with its internalization and degradation in acidic compartments. Finally, the observed CD4 downregulation induced by PC liposomes in human primary macrophages reduced the entry of both single-cycle replication and replication competent R5 tropic HIV-1. Altogether, these results show that PC liposomes reduce HIV entry in human macrophages and may impact HIV pathogenesis by lowering the viral reservoir.

## Introduction

Despite the efforts and improvements in antiretroviral therapy (ART), human immunodeficiency virus type-1 (HIV-1) infection still remains one of the most relevant public health emergencies, recording approximately 37.7 million worldwide people living with HIV-1 (PLWH) and 1.5 million new infections in 2020 (1). The combined pharmacological action of drugs, interfering with different stages of the viral replication cycle, represents a valid strategy to counteract HIV-1 infection. As a result, a number of antiretroviral drugs have been developed, approved and are currently in use: i) nucleoside reverse transcriptase inhibitors (NRTIs) and non-nucleoside reverse transcriptase inhibitors (NNRTIs), which impede viral DNA synthesis; ii) integrase strand transfer inhibitors (INSTIs), which block HIV-1 DNA integration into the host cell genome; iii) protease inhibitors (PIs), which prevent the formation of mature and infectious virions by blocking the cleavage of viral precursor proteins; and iv) inhibitors of virus fusion and entry (2–4). An essential step that allows HIV-1 infection is certainly the capability of the virus to interact with and enter target cells. HIV-1 avails of envelope glycoprotein gp120, which first binds to the cellular receptor CD4 and then to a second co-receptor, allowing gp120 conformational change and the fusion led by viral gp41 between the host membrane and viral envelope with subsequent virus entry. The kind of co-receptor used determines the viral tropism, with viruses using chemokine receptor type 5 (CCR5) named R5 HIV-1, viruses using C-X-C Motif Chemokine Receptor type 4 (CXCR4) named X4 HIV-1, and viruses with dual tropism named R5X4 HIV-1 (5). Among drugs that interfere with the first steps of HIV-1 infection, we may identify i) CCR5 antagonists that imped the interaction between co-receptor and gp120, ii) gp120 antagonists that bind to the virus, preventing the association with the host CD4 receptor, iii) gp41 antagonists that inhibit the fusion with the host cell membrane, and iv) CD4 antagonists, monoclonal antibodies that block HIV-1 entry into target cells (6). However, the high genetic variability of HIV-1 and the rapid generation of drug-resistant viral variants often hinder the action of antiviral drugs that directly target essential viral components (7, 8). On these grounds, the modulation on host cell target of the expression of receptors exploited by the virus may represent an attractive novel strategy to interfere with its entry, preventing HIV-1 infection.

CD4, a transmembrane glycoprotein of 55–60 kDa belonging to the immunoglobulin superfamily, is composed of four extracellular domains, a transmembrane domain, and a cytoplasmic tail, and exerts a key role in HIV-1 pathogenesis, being the first receptor with which the virus interacts. CD4 plays a crucial role in T-cell development, maturation, and activation as it is involved in stabilizing interactions between TCR and the peptide–MHC II complex and mediates intracellular signal transduction. CD4 is also present on cells of myeloid lineage, such as monocytes and macrophages, although at lower levels than lymphocytes (9). It has been reported that ligation of CD4 on monocytes triggers differentiation into macrophages, but the role of the receptor in differentiated macrophages remains controversial (10). Macrophages represent the second most HIV-1-infected cellular type, are scarcely susceptible to the cytopathic effect of the R5 HIV-1 strains (11) and can considerably contribute to viral burden (12) by supporting plasma viremia after CD4^+^ T-cell depletion (13, 14). Furthermore, viruses may be stored for a long time within myeloid cells in intracellular vesicles named virus-containing compartments (VCC), which may provide a protective environment for viruses, since they are not readily accessible by immune effector molecules and play a role as viral reservoirs for HIV-1 in infected individuals (13–15).

Abnormalities in the phospholipid composition of bronchoalveolar lavage (BAL) have been previously reported during HIV infection, with phosphatidylcholine (PC) content being decreased at all stages of disease (16, 17). Moreover, phospholipids, including PC, not only take on a structural function but are also implicated in many cellular processes, such as vesicle trafficking, signal transduction, and down-regulation of membrane protein receptors (18, 19). On these grounds, we evaluated the capability of PC liposomes to down-modulate the expression of the CD4 receptor in human primary type-1 macrophages and studied the mechanisms underlying this event. Finally, we evaluated the effectiveness of these liposomes to interfere with viral entry in an *in vitro* model of HIV-1 infection.

## Method

### Cell Cultures

Primary cultures were prepared as previously described (20). Briefly, peripheral blood mononuclear cells (PBMCs) were isolated by Ficoll (Cedarlane) density gradient centrifugation. Monocytes were sorted using anti-CD4 monoclonal antibodies conjugated to magnetic microbeads (Miltenyi Biotec) according to the instructions of the manufacturer. Purified monocytes were then suspended in RPMI 1640 supplemented with 10% fetal bovine serum (FBS), 2 mM L-Glutamine and 5 μg/ml gentamicin (complete medium), seeded in 24-well plates (5 × 10^5^ cells/well), 48-well plates (3 × 10^5^ cells/well), or in 96-well plates (2 × 10^5^ cells/well), and cultured for 5 days at 37°C with 5% CO_2_ in the presence of 35 ng/ml Granulocyte Macrophage-Colony Stimulating Factor (GM-CSF, R&D System) to obtain type-1 oriented macrophages (21), whose phenotype (22) was confirmed in **Figure S1**. Lymphocyte fractions were suspended in complete medium and seeded in 48-well plates (10^6^ cells/well) for immediate stimulation with liposomes.

### Liposome Preparation

Liposomes were produced as previously described (23, 24). In brief, L-α-phosphatidylcholine (PC) (Avanti Polar Lipids) in anhydrous dodecane (Sigma-Aldrich) was used as an inner monolayer lipid at a concentration of 0.05 mg/ml. For the outer monolayer, the lipid L-α-phosphatidylserine (PS) (Avanti Polar Lipids) or PC was used in a 99:1 dodecane–silicone solution to obtain a final concentration of 0.05 mg/ml. Thereafter, liposomes were prepared by adding 2 ml of outer monolayer lipid suspension to more than 3 ml of complete medium. Finally, 100 μl of the inner monolayer lipid suspension was added to over 2 ml of lipid phase, and the samples were centrifuged at 120×*g* for 10 min. After centrifugation, liposomes were collected in an aqueous phase using a 5 ml syringe with a 16-gauge stainless steel needle, to produce PS outside/PC inside (PS/PC) and PC outside/PC inside (PC/PC). The liposomes were then quantified by a flow cytometer, FACSCalibur (Becton Dickinson), allowing the quantification of monodispersed vesicles >0.2  μm in diameter.

### Cell Stimulation

Lymphocytes fraction and type-1 macrophages were stimulated with different liposome formulations, used at the ratio of 1:1 (liposome:cell) for 18 h or, in the case of time course kinetics, as indicated in each figure legend. Where indicated, 10 nM RO 31-8220 (Sigma-Aldrich), a general inhibitor of protein kinase C (PKC), and 100 nM Gö 6976 (Sigma-Aldrich), an inhibitor of Ca^2+^-dependent PKC, were added, according to the instructions of the manufacturer, 30 min before liposome stimulation. Finally, in several experiments, 10 nM concanamycin A (Santa Cruz Biotechnology), was used to block the phagosome maturation, as described (24, 25).

### Flow Cytometry

Flow cytometry analysis was performed using FITC-labeled anti-CD3 (Clone OKT3, BioLegend), PE labeled anti-CD195 (CCR5) (Clone REA245, Miltenyi Biotec), PerCP-Vio700 labeled anti-CD4 (Clone VIT4, Miltenyi Biotec) monoclonal antibodies, for cell surface staining, and APC labeled anti-CD4 (Clone VIT4, Miltenyi Biotec) monoclonal antibodies, for intracellular staining. Briefly, cell surface staining was performed by incubating cells for 30 min at 4°C with FITC-labeled anti-CD3, PE-labeled anti-CD195, and PerCP-Vio700-labeled anti-CD4 monoclonal antibodies. In experiments requiring intracellular staining of CD4, cells were then washed, fixed with 4% paraformaldehyde, permeabilized with phosphate-buffered saline (PBS) supplemented with 2% FBS and saponin 0.5% (Sigma-Aldrich) and then stained with APC labeled anti-CD4 monoclonal antibodies for 30 min at 4°C. Flow cytometry analysis was carried out using a FACSCalibur flow cytometer (Becton Dickinson) and data were processed using FlowLogic software (Miltenyi Biotec). Data are shown as percentage of positive cells and Median Fluorescence Intensity (MFI). Lymphocyte CD4 and CCR5 values were obtained after gating on CD3-positive cells.

### Western Blotting

Macrophages (5 × 10^5^ cells/well) were stimulated with PC liposomes for 5 min, 10 min, 20 min, 30 min, 1 h, and 18 h. Cells were harvested using cold PBS, centrifuged twice at 558×*g* for 5 min and stored to −80°C. Cell pellets (2 × 10^6^ cells) were lysed in RIPA buffer (50 mM Tris–HCl, pH 8.0, 150 mM NaCl, 12 mM deoxycholic acid, 0.5% Nonidet P-40) containing 1% (v/v) protease and phosphatase inhibitor cocktail (Sigma-Aldrich). Five μg of proteins were loaded on SDS-PAGE and subjected to Western blotting. Nitrocellulose membranes were incubated with CD4 (sc-19641, Santa Cruz Biotechnology) or phospho-CD4 (SAB4504134, Sigma-Aldrich) primary antibodies at a 1:1,000 dilution. Staining with GAPDH antibody (sc-47724, Santa Cruz Biotechnology, TX, USA) was used as a loading control. Successively, membranes were incubated with the appropriate horseradish peroxidase-conjugated secondary antibodies. After incubation of the membranes with ECL Selected Western Blotting Detection Reagent (GE Healthcare, Pittsburgh, PA, USA), immunoreactive bands were detected by a FluorChem FC3 System (Protein-Simple, San Jose, CA, USA). Densitometric analyses of the immunoreactive bands were performed by the FluorChem FC3 Analysis Software.

### Infection by a Pseudotyped HIV-1 Reporter Virus

A pseudotyped HIV-1 luciferase virus able to complete a single round of infection was used (26). To produce recombinant HIV-1 virions, human embryonic kidney (HEK) 293 cells were cotransfected with 12 µg of pSVC 2.1 vpr+ vpu+ nef+ ΔBglII rev- Luc, 5 µg of pREV, and 6 µg of pSVIII plasmid containing HIV-1 Ba-L envelope glycoprotein or pSVIII-VSV-G (vesicular stomatitis virus envelope glycoprotein) or pSVIII-KSenv (a nonfunctional envelope glycoprotein) with a ProFection mammalian transfection system-calcium phosphate (Promega). Cell supernatants carrying progeny pseudotyped virions were harvested 48 h after transfection, clarified, filtered, aliquoted, and stored at −80°C. The virus production in the cell-free supernatants was normalized for reverse transcriptase (RT) activity as previously described (27) and used to infect TZM-bl cells for titration. After 48 h of incubation, 100 µl of culture medium was removed from each well, 100 µl of Bright-Glo reagent (Promega) was added to the cells, and then luminescence was measured by considering Relative Luminescence Units (RLU) of >2.5 times a positive result. For infection, type-1 macrophages (5 × 10^5^ cells/well) in 24-well plates were stimulated with PC liposomes for 18 h and then were washed twice to remove internalized liposomes. The expression of CD4 and CCR5 was examined by flow cytometry. Then, macrophages were infected overnight with 1 × 10^4^ TCID50 units of virus. After infection, cells were washed twice to remove the unbound viruses and cultured in complete medium in the presence of 33 ng/ml GM-CSF for 6 days. Cells were harvested, washed once with PBS, and lysed in 100 µl of lysis reagent (Promega) and the presence of luciferase was quantified by measuring RLU using a Victor Nivo Perkin Elmer.

### Infection With Replication Competent R5-Tropic HIV-1 81A Strain

Type-1 macrophages (3 × 10^5^/well) were pre-treated with PC liposomes for 18 h, washed and then infected with the R5-tropic HIV-1 81A strain at a concentration of 10,000 pg/ml p24 per 10^6^ cells. The infection was performed for 2 h in a 48-well plate at a volume of 500 µl. After infection, cells were washed twice and cultured in complete medium for 4 days.

### Quantification of HIV-DNA by ddPCR

A QX200 Droplet Digital PCR System (ddPCR, Bio-Rad) was used for the quantification of total HIV-1 DNA in type-1 macrophages. Total DNA was extracted from a pellet of cells 4 days post-infection, in both treated and untreated HIV-1 infected macrophages and for negative control in non-infected macrophages, by using the Allprep^®^ DNA/RNA mini kit (Qiagen, Germany) according to the instruction of the manufacturer. Total HIV-DNA was quantified by ddPCR using a home-made assay, targeting the 5’-LTR of HIV-1 (28). DNA copies were normalized to cell number according to the quantification obtained from the Albumin-based ddPCR copy number assay: Alb, Human (Bio-Rad, Pleasanton, California, USA) and, thus, reported as HIV-DNA copies/3 × 10^5^ cells (29).

### Statistical Analysis

Comparison between groups was done using Student’s *t*-test, as appropriate for normally distributed data. The Wilcoxon matched pairs signed rank test was performed for data that were not normally distributed. Analysis of Western blotting data has been performed by means of the Kruskal–Wallis Uncorrected Dunn’s test to estimate the differences between samples. p-values lower than  0.05 were considered statistically significant.

### Ethics Statement

Buffy coats from anonymized healthy donors who gave their written informed consent to donate the non-clinically usable components of their blood for scientific research were obtained from Policlinico Tor Vergata, in Rome. All procedures described here were authorized by the Ethics committee of the University of Rome “Tor Vergata” (R.S. # 16/20).

## Results

### Phosphatidylcholine Liposomes Downregulate CD4 Expression on Type-1 Macrophages

**Figure 1:**
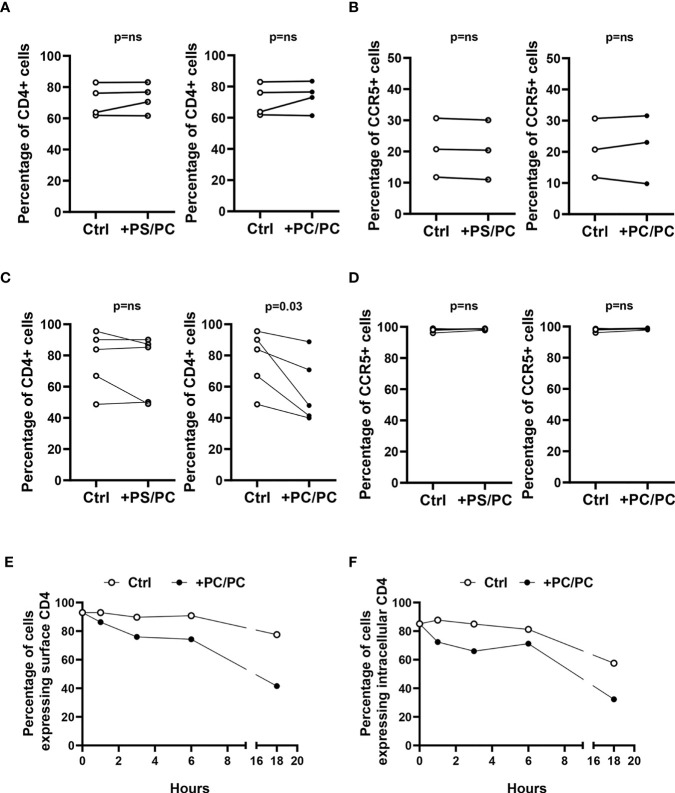
Downregulation of CD4 expression on type-1 macrophages. **(A, B)** Lymphocytes (10^6^ cells/0.5 ml) were stimulated or not with liposome formulations at the ratio 1:1 (liposome:cell) for 18 h. Cells were collected, stained with anti-CD3-FITC, anti-CD4-PerCP-Vio700, and anti-CCR5-PE and analyzed by flow cytometry. Figure shows the percentage of CD4 positive cells **(A**, n = 4**)** and CCR5 positive cells **(B**, n = 3**)** of CD3 positive cells. p-value was obtained by one-sided Wilcoxon matched-pairs signed rank test (p = ns, not significant). **(C, D)** Type-1 macrophages (5 × 10^5^ cells/ml) were stimulated or not with liposome formulations at the ratio 1:1 (liposome:cell) for 18 h. Cells were collected, stained with anti-CD4-PerCP-Vio700 and anti-CCR5-PE and analyzed by flow cytometry. Figure shows the percentage of CD4 **(C**, n = 5**)** and CCR5 **(D**, n = 5**)** positive cells. p-value was obtained by one-sided Wilcoxon matched-pairs signed rank test (p = ns, not significant). **(E, F)** Macrophages (5 × 10^5^ cells/ml) were stimulated or not with PC liposomes at the ratio 1:1 (liposome:cell) for 1, 3, 6, and 18 h and then collected. Cells were stained with anti-CD4-PerCP-Vio700, fixed, permeabilized, stained with anti-CD4-APC and finally analyzed by flow cytometry. Data show the percentage of cells expressing surface CD4 **(E)** and intracellular CD4 **(F)** and are representative of five experiments performed on cells from different healthy donors.

CD4 downregulation in monocyte-derived macrophages (MDM) has previously been reported to be mediated by PKC, which induces serine phosphorylation at the intracytoplasmic tail of CD4 and targets the receptor to intracellular degradative compartments (30). As phosphatidylcholine (PC) may be metabolized by different phospholipases, whose products may directly or indirectly activate PKC (31), we evaluated the capability of PC liposomes to modulate the expression of the CD4 receptor on type-1 primary macrophages. For this aim, we generated liposomes composed entirely of PC (PC/PC) and liposomes composed of PC at the inner leaflet and of phosphatidylserine (PS) at the outer leaflet (PS/PC), in order to resemble apoptotic bodies and facilitate their internalization within macrophage target cells. The liposome preparations were preliminarily tested in flow cytometry for size distribution by comparing their forward scatter parameters (FS) with the FS of commercially available beads of known diameter. The liposome formulations reported herein show a dimensional distribution between 0.8 and 6 µm diameter (**Figure S2**). Liposomes were then used to stimulate for 18 hours human type-1 primary macrophages and lymphocytes. Both cell types were then analyzed for the surface expression of CD4 and CCR5, both in terms of percentage of positive cells and Median Fluorescence Intensity (MFI). The treatment with liposome formulations did not modulate CD4 and CCR5 expression on T lymphocytes (**Figures 1A, B**, **S3**). In contrast, the stimulation of macrophages with PC/PC led to a significant reduction of CD4 compared to unstimulated control cells (**Figures 1C**, **S4**), whereas no effect was observed either in terms of CCR5 expression (**Figures 1D**, **S4**) or in terms of cytotoxicity (**Figure S5**). To investigate the kinetics of CD4 down-modulation, we monitored the intracellular and cell surface expression of the receptor in macrophages treated or not with PC/PC at different time points up to 18 h. Results show that the treatment with PC/PC induced a progressive reduction of the membrane receptor, reaching its highest decrease after 18 h of stimulation (**Figure 1E**). A similar kinetic of progressive reduction could be observed by evaluating the intracellular CD4 expression (**Figure 1F**), suggesting its rapid degradation into lytic compartments. Finally, a significant down-modulation of both surface and intracellular CD4 expression levels was then observed by analyzing cumulative experiments performed on cells from 5 healthy donors after 18 h of PC liposome treatment (**Figure S6**).

### Phosphatidylcholine Liposomes Induce CD4 Down-Modulation by Promoting Its Intracellular Degradation by Serine Phosphorylation

**Figure 2:**
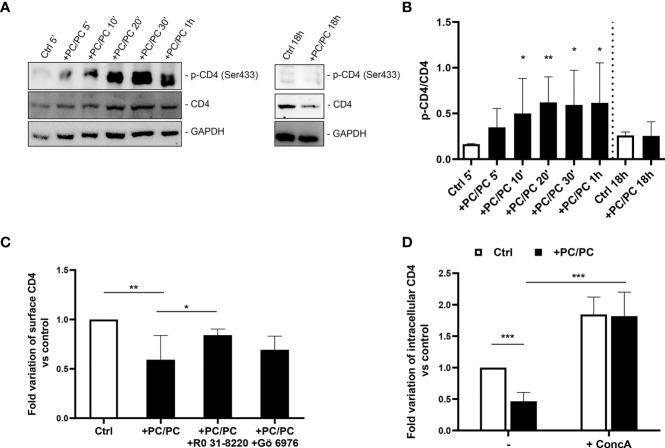
Downregulation of CD4 expression involves acidic compartments by serine phosphorylation. **(A, B)** Macrophages (5 × 10^5^ cells/ml) were stimulated or not with PC liposomes at the ratio 1:1 (liposome:cell) for 5, 10, 20, 30 min, 1, and 18 h. Cells were collected (2 × 10^6^), stored at −80°C and then analyzed by western blotting, following lysis. Figure shows a representative Western blotting **(A)** and the densitometric analysis of the levels phosphorylated CD4 on serine 433 (p-CD4 Ser433) normalized to CD4 basal signal **(B)** shown as mean ± Standard Deviation (SD) of values obtained from four different healthy donors. GADPH was used as loading control. *p <0.05 and **p <0.01 by Kruskal–Wallis Uncorrected Dunn’s test, in comparison with non-stimulated control. **(C)** Macrophages (5 × 10^5^ cells/ml) were pre-treated or not with 10 nM RO 31-8220 and 100 nM Gö 6976 for 30 min and then stimulated or not with PC liposomes at the ratio 1:1 (liposome:cell) for 18 h. Cells were collected, stained with anti-CD4-PerCP-Vio700 and analyzed by flow cytometry. Data are shown as mean ± SD of fold variation of percentage of CD4 positive cells calculated by normalizing the percentage of CD4 positive cells obtained from each healthy donor on their own non-stimulated control (n = 5). *p <0.05 and **p <0.01 by Student’s *t*-test. **(D)** Macrophages (5 × 10^5^ cells/ml) were pre-treated or not with 10 nM concanamycin A (ConcA) for 30 min, stimulated or not with PC liposomes at the ratio 1:1 (liposome:cell) for 18 h and then were collected. Cells were stained with anti-CD4-PerCP-Vio700, fixed, permeabilized, stained with anti-CD4-APC and finally analyzed by flow cytometry. Data are shown as mean ± SD of fold variation of percentage of positive cells expressing intracellular CD4 calculated by normalizing the percentage of CD4 positive cells obtained from each healthy donor on their own non-stimulated control (n = 5). ***p <0.0001 by Student’s *t*-test.

It has been previously reported that CD4 internalization may depend upon serine phosphorylation at the intracytoplasmic tail of the receptor (32, 33). On these grounds, we analyzed the levels of phosphorylated CD4 protein on serine 433. A progressive increase in phosphorylated CD4 was observed after PC liposome treatment that was significant starting from 10 min after stimulation, and such phosphorylation was upstream of receptor degradation (**Figures 2A, B** **)**. As it has been previously reported that PKC may be responsible for CD4 phosphorylation (30), we investigated the modulation of CD4 on the macrophage cell surface either after aspecific inhibition of all PKCs (by using RO 31-8220 inhibitor) or after specific inhibition of Ca^2+^-dependent PKCs (using Gö 6976 inhibitor). Results show that only RO 31-8220 inhibitor significantly recovered the expression of CD4 in macrophages following PC liposome treatment in terms of both % of CD4 expressing cells (**Figure 2C**) and MFI (**Figure S7**), excluding the involvement of Ca^2+^-dependent PKCs.

As membrane CD4 down-modulation is not associated with its intracellular accumulation, we hypothesized that receptor phosphorylation may represent a signal for its degradation in acidic compartments. To test this hypothesis, we stimulated macrophages with PC liposomes in the presence or not of concanamycin A, a specific inhibitor of v-ATPase that raises the pH of acidic organelles, and monitored the expression of intracellular CD4. Results show that concanamycin A significantly increased intracellular CD4-expression in terms of both % of CD4 expressing cells (**Figure 2D**) and MFI (**Figure S8**).

### Phosphatidylcholine Liposomes Reduce HIV Entry in Human Primary Macrophages

**Figure 3:**
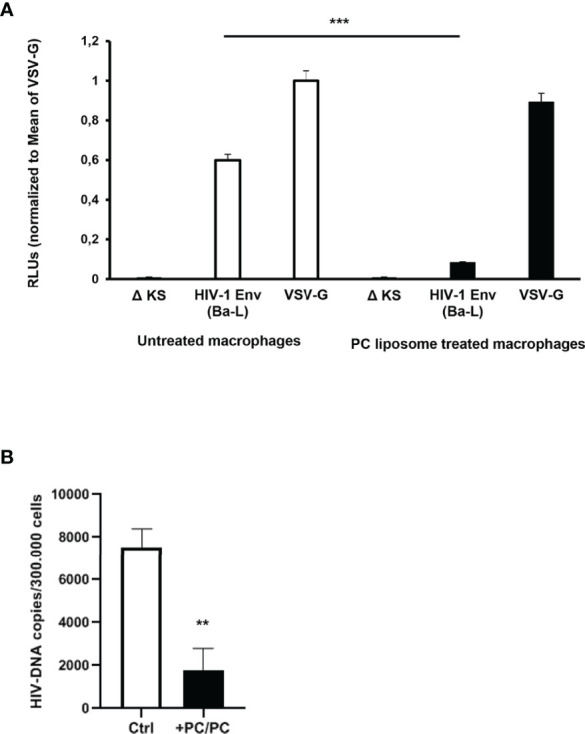
Down-modulation of CD4 expression in PC liposomes pre-treated macrophages reduces HIV-1 entry. **(A)** Macrophages (5 × 10^5^ cells/ml) were pre-treated or not with PC liposomes for 18 h at the ratio 1:1 (liposome:cell), washed and then infected with HIV-1 Env (Ba-L)- or VSV-G-pseudotyped luciferase-encoding HIV-1. After infection, cells were washed, cultured for 6 days, and infection was quantified by luciferase activity. RLUs were normalized to the value for luciferase obtained in the VSV-G-pseudotyped virus-infected cells. Mean relative luciferase activities ± SD are shown (three independent experiments performed in quadruplicate on cells from three different healthy donors; ***p <0.0001 by Student’s *t*-test). A nonfunctional envelope (HIV-1-ΔKS) was used as a negative control to determine the background levels of luciferase activity. **(B)** Type-1 macrophages (3 × 10^5^ cells/ml) were pre-treated or not with PC liposomes for 18 h at the ratio 1:1 (liposome:cell), washed and then infected with replication competent R5 HIV-1 81A strain. After infection, cells were washed, cultured for 4 days, and intracellular HIV-DNA was quantified by ddPCR. Total amount of HIV-DNA was normalized on cell number obtained by the quantification of Albumin. Results are shown as mean HIV-DNA values ± SD of the values obtained from 4 replicates and are representative of experiments performed on cells from two healthy donors (**p = 0.0005 by Student’s *t*-test).

As the first interaction between HIV-1 and a host cell occurs between viral gp120 and the CD4 receptor expressed on the surface of target cells, we analyzed the possibility that PC liposomes can induce macrophages to be less susceptible to HIV entry. For this aim, macrophages were pre-treated or not with PC liposomes for 18 h and then infected with a single-round HIV-1 luciferase reporter virus pseudotyped with either HIV Ba-L env (CCR5-tropic) or vesicular stomatitis virus G (VSV-G) (CD4/CCR5-independent) glycoproteins (26). Pre-treatment with PC liposomes significantly inhibited the luciferase activity in macrophages infected with HIV-1 (Ba-L Env) but not in those infected with HIV-1 (VSV-G) (**Figure 3A**), indicating that PC liposome-mediated CD4 downregulation reduced HIV-1 infection in type-1 macrophages at entry level. In all experiments, the envelope defective virus ΔKS failed to infect macrophages (**Figure 3A**). Finally, type-1 macrophages were also infected with the replication competent HIV-1 CCR5-tropic 81A strain. Total HIV-DNA was extracted from infected and uninfected cells and quantified by ddPCR. Pre-treatment with PC liposomes significantly decreased HIV-DNA levels (**Figure 3B**), with an inhibition of about 70%, confirming that PC liposome mediated CD4 downregulation reduced type-1 macrophage infection at entry level.

## Discussion

Since the early epidemic, lung opportunistic infections have been one of the leading causes of mortality in PLWH and, although the introduction of ART changed the spectrum of pulmonary complications, lung infections still remain a frequent cause of mortality (34–36). The development of ART has certainly improved the life expectancy of PLWH, controlling viral spread and preventing the progression of acquired immunodeficiency syndrome (AIDS). However, ART does not eradicate the virus as it may persist indefinitely within the cellular reservoir, which is responsible for rapid viral rebound in infected individuals after ART interruption. Along with CD4^+^ T lymphocytes, macrophages are the main target of HIV, supporting viral persistence and replication even in subjects undergoing ART or with massive CD4^+^ T-cell depletion (37). Macrophages represent a viral reservoir, due to their long life span and resistance to viral cytopathic effect and simultaneously, they are an important vehicle for viral spread for their ubiquitous distribution, including, gut, semen, lung, gut-associated lymphoid tissue, brain, liver, urethra, and lymph nodes (37–40). Macrophages are a heterogeneous cell population, which may range from cells with pro-inflammatory properties and efficient antigen presentation capability, named “classically activated” type-1 macrophages, to cells which are rather involved in the resolution of inflammation, called “alternatively activated” type-2 macrophages (41, 42). The balance between these two cell types is fundamental for correct immune response and largely depends on the tissue, the local cytokine microenvironment and differences in infectious stimuli (43). Due to their excellent capability to clear the engulfed pathogens, type-1 macrophages are the first cells that accumulate during the acute phase of viral and bacterial infection (44, 45). However, the alteration of microbicidal responses exhibited by HIV-infected macrophages (46) may impair their capability to counteract pulmonary opportunistic infections. Moreover, small alveolar macrophages, showing a type 1-like phenotype (47), are preferentially infected by HIV (48) and may represent long-lived cellular reservoirs for the virus within the lung, where the large number of cells in close proximity provides fertile grounds for cell-to-cell spread of HIV (49).

On these grounds, this work aims to exploit the possibility of limiting HIV entry and hence viral reservoir establishment in type-1 macrophages, considered key cells in pulmonary HIV-related complications occurring during opportunistic infections.

Previous studies have reported that CD4 downregulation may be the result of tissue localization and of the local microenvironment and that such reduction is correlated with a lower propensity to HIV infection (50). Moreover, it has been reported the possibility to down-modulate surface CD4 expression without affecting co-receptors using different types of stimuli, such as microRNA (51), proteins (52) and T-cell-derived soluble factors (30). The results reported here show that PC liposomes can down-modulate the expression of CD4 in human type-1 macrophages but not in helper T cells, without affecting cell viability, and hence can interfere with the initial steps of HIV-1 infection.

Additionally, our results show that the CD4 downregulation is induced by liposomes entirely constituted by PC and not by asymmetric PS/PC liposomes. These results suggest that the phagocytosis process, induced by the recognition of PS, may hinder the availability of PC for the downstream CD4 downregulation. Moreover, a possible association between the internalization of CD4 and the activity of PKC has been reported (32, 33). Our results are coherent with the involvement of Ca^2+^-independent PKCs, as the inhibition of total PKCs, but not the specific inhibition of Ca^2+^-dependent PKCs, significantly rescues membrane CD4 expression. The mechanisms by which PC liposomes activate PKC are still unknown and may be mediated by the possible involvement of different phospholipases, whose products may directly or indirectly activate the enzyme (31).

The results reported here also show that PKC-dependent CD4 down-modulation is associated with phosphorylation of serine 433 at the intracytoplasmic tail of CD4, which precedes its progressive loss. Interestingly, the addition of concanamycin A, which inhibits vATPase-mediated vacuole acidification (53), significantly increases the levels of intracellular CD4, supporting the hypothesis that PKC, by phosphorylating CD4, targets it to degradative acid compartments. These results are coherent with the previously reported results showing that stimulation of macrophages with T-cell-mediated soluble factors induced CD4 downregulation by its phosphorylation and its targeting to an intracellular compartment destined for acidification and degradation (30). Moreover, PC liposome-induced CD4 downregulation significantly inhibits the entry of the R5 single cycle replication HIV-1 Ba-L strain into human macrophages. Simultaneously, PC liposome treatment does not influence the VSV-G entry, whose internalization is independent by CD4 or CCR5, supporting the hypothesis that PC liposomes affect CD4-dependent viral entry. These results were further confirmed using the replication competent R5 HIV 81A strain. The biological impact of CD4 downregulation, induced by PC liposomes, on HIV-1 entry confirms previous studies showing that small variations in CD4 expression in macrophages may have a disproportionate effect on entry by R5 HIV viruses (51, 54, 55).

The lungs are the principal target of human immunodeficiency virus (HIV)-associated complications pulmonary diseases (56). Additionally, pulmonary co-infections promote an inflammatory microenvironment that facilitates both HIV-1 infection and replication in the lung by favoring cell-to-cell viral spread (57). On the basis of the results reported herein, a possible therapeutic strategy based on the aerosolic administration of PC liposomes may reduce the susceptibility to HIV infection of inflammatory type-1 macrophages and lower the viral reservoir in the lung. Such a reduction may in turn contribute to limit the viral transmission and consequent depletion of pathogen-specific CD4^+^ T cells, thereby preventing the reactivation of opportunistic pathogens during HIV infection (58).

## Data Availability Statement

The original contributions presented in the study are included in the article/**Supplementary Material**. Further inquiries can be directed to the corresponding author.

## Ethics Statement

The studies involving human participants were reviewed and approved by the Ethics Committee of University of Rome “Tor Vergata” (R.S. #16/20). The patients/participants provided their written informed consent to participate in this study.

## Author Contributions

FDS, AB, MMDA, FC-S, MF, SA, and KA contributed to the conception and design of the study. FDS, ABL, SV, LD, TO, PS, and LHDA contributed to data acquisition. FDS, AB, NP, FC-S, and MF, participated in data analysis and manuscript writing. All authors listed have made a substantial, direct, and intellectual contribution to the work and approved it for publication.

## Funding

This work was supported by i) the Italian Foundation for Multiple Sclerosis, grant #2016/R/22; ii) the Horizon 2020, grant #643558; iii) the Italian Cystic Fibrosis Research Foundation, FFC #21/2019; and iv) the PRIN (Progetti di Rilevante Interesse Nazionale) Grant 2017M8R7N9_004 from the MIUR, Italy.

## Conflict of Interest

The authors declare that the research was conducted in the absence of any commercial or financial relationships that could be construed as a potential conflict of interest.

## Publisher’s Note

All claims expressed in this article are solely those of the authors and do not necessarily represent those of their affiliated organizations, or those of the publisher, the editors and the reviewers. Any product that may be evaluated in this article, or claim that may be made by its manufacturer, is not guaranteed or endorsed by the publisher.
