# Supplementary material for: Phosphatidylcholine Liposomes Down-Modulate CD4 Expression Reducing HIV Entry in Human Type-1 Macrophages
Source: Front Immunol. 2022 May 19;13:830788. doi: 10.3389/fimmu.2022.830788 (PMC9160374; doi:10.3389/fimmu.2022.830788)
Supplement: Supplementary file 1 [file DataSheet_1.pdf]

Supplementary Material

Supplementary Methods

Analysis of type-1 macrophages (M1) phenotype. Peripheral blood mononuclear cells (PBMC) from buffy coat were isolated by Ficoll density gradient and monocytes were then positively sorted using anti-CD14 monoclonal antibodies conjugated to magnetic microbeads (Miltenyi Biotec), according to manufacturer's instructions. Monocytes were then suspended in complete medium and incubated for a further 5 days in 24-well plates at the concentration of 10^6 cells/ml in the presence of GM-CSF (35 ng/mL) (Miltenyi Biotec) to get differentiated M1. The M1 phenotype was then confirmed by flow cytometry (FACSCelesta, BD) after staining cells with PE-Cy™7 labelled anti-CD45, BV786 labelled anti-CD14, BB515 labelled anti-CD86, BB700 labelled anti-CD282 (all by BD) and labelled APC anti-HLA-DR (Santa Cruz) monoclonal antibodies and by Interleukin- 1 β (IL-1β) and Interleukin-10 (IL-10) release in the supernatant by DuoSet® ELISA Development Systems (R&D), used according to the manufacturer's instructions.

Dimensional analysis of liposomes by flow cytometry. The average diameter of PS/PC and PC/PC was assessed comparing the median forward scatter with commercially available microbeads (Mb) with the diameter of 0.8 (Sigma-Aldrich), 2, 4 and 6 µm (Thermo Fisher), by using the flow cytometer Celesta (BD) and analysed by the FlowLogic software (Milteny Biotec). Each sample is displayed as a proportion of its highest value, which is shown at 100%.

Cell viability assay. Type-1 macrophages ($2x10^5$ cells/200 µl) were stimulated with liposomes and cell viability was evaluated by the MTT Cell Proliferation Assay Kit (Molecular Probe), used according to the manufacturer's instructions. The assay is based on the cleavage of the yellow tetrazolium salt MTT (3-(4,5-Dimethylthiazol-2-yl)-2,5-diphenyltetrazolium bromide) to purple formazan crystal in metabolically active cells. The formazan is then solubilized, and the concentration determined by optical density at 540 nm. The assay is sensitive with the colorimetric signal proportional to the viable cell number. As negative control, macrophages were treated with 0.1% saponin at 37˚C for 30 min (data not shown).

Supplementary Figures

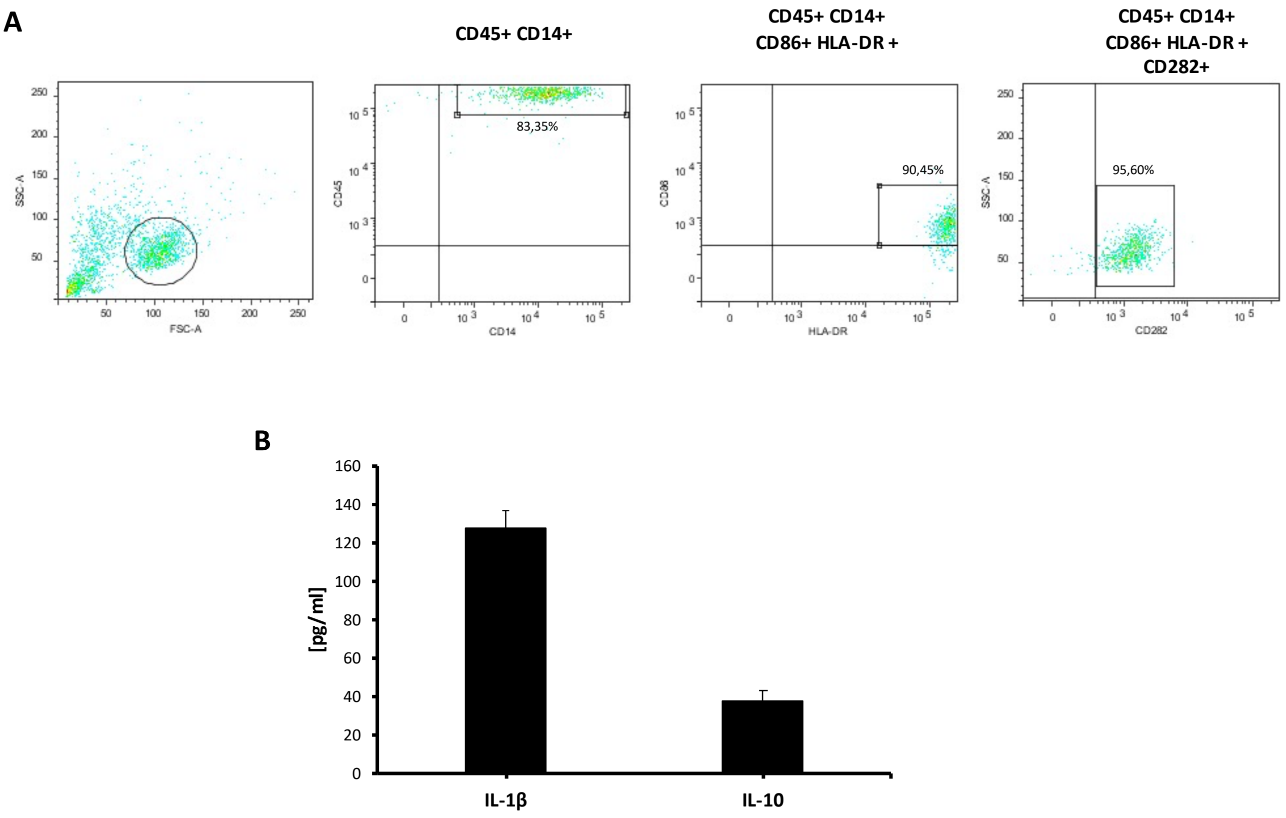

Figure S1. Phenotypic characterization of type-1 macrophages. Purified monocytes were cultured for 5 days with 35 ng/ml of GM-CSF and then analyzed in terms of membrane phenotype (A) and cytokine release (B). **(A)** Plots show the gating strategy and the percentage of cells expressing CD45, CD14, CD86, HLA-DR and CD282, used as specific type 1 macrophage receptors. Results are representative of experiments with cells from two different healthy donors. **(B)** IL-1β and IL-10 release in the supernatants was analyzed and are shown as the mean $\pm$ SD of the values obtained from triplicate cultures and are representative of experiments with cells from two different healthy donors.

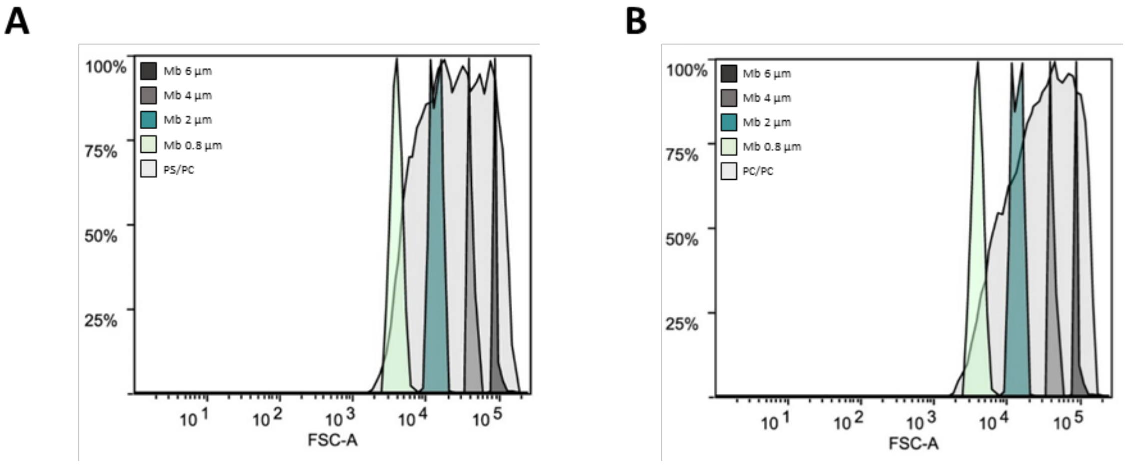

Figure S2. Dimensional analysis of liposomes. Forward scatter (FSC-A) distribution of beads of 0.8, 2, 4 and 6 µm and of PS/PC **(A)** and PC/PC **(B)** liposomes.

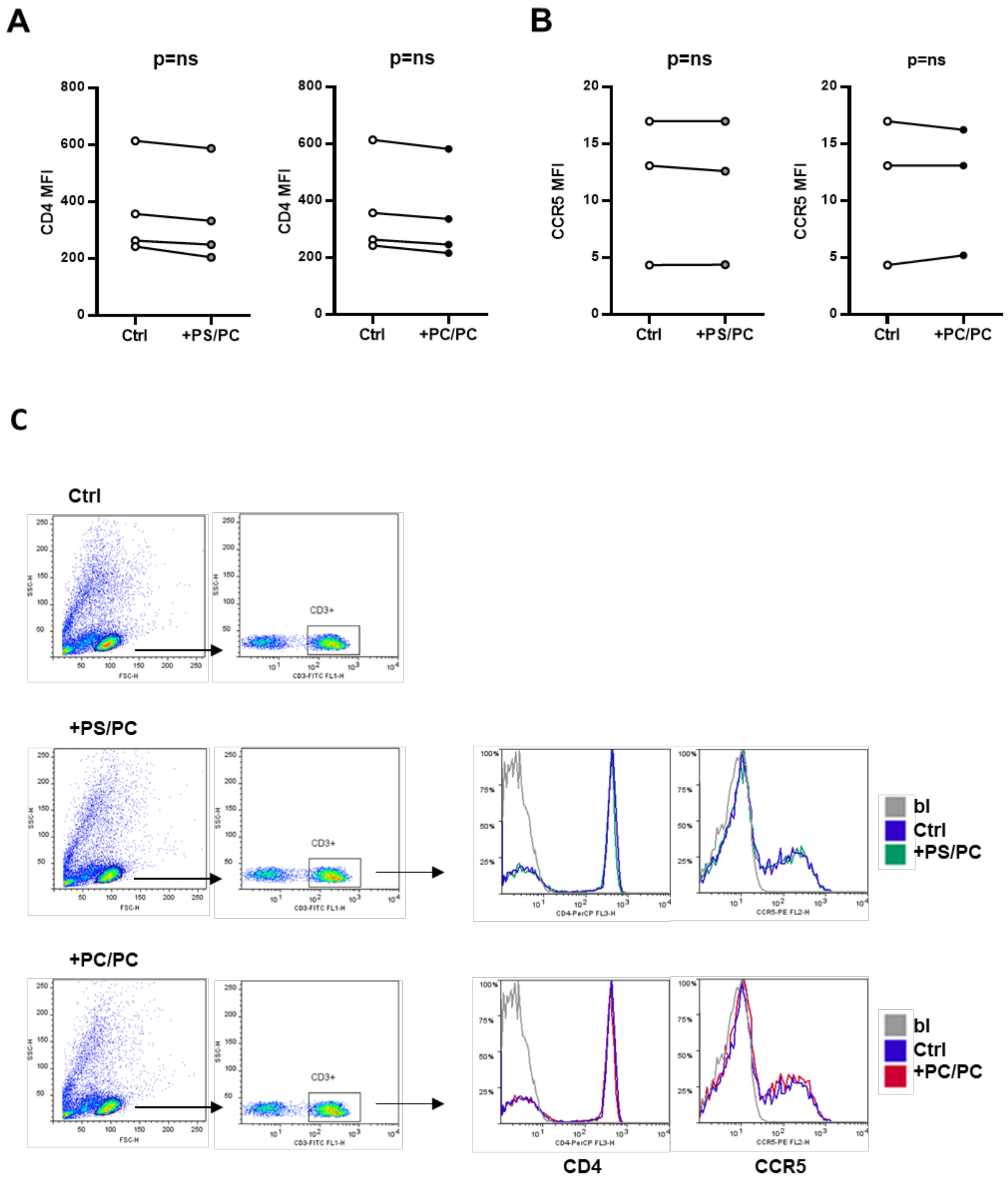

Figure S3. CD4 and CCR5 expression on lymphocytes. (A, B) Median Fluorescence Intensity (MFI) of membrane CD4 **(A**, n=4**)** and membrane CCR5 **(B**, n=3**)** on lymphocytes (CD3 positive cells). **(C)** Representative dot plots and overlays. p value was obtained by one-sided Wilcoxon matched-pairs signed rank test (p=ns, not significant).

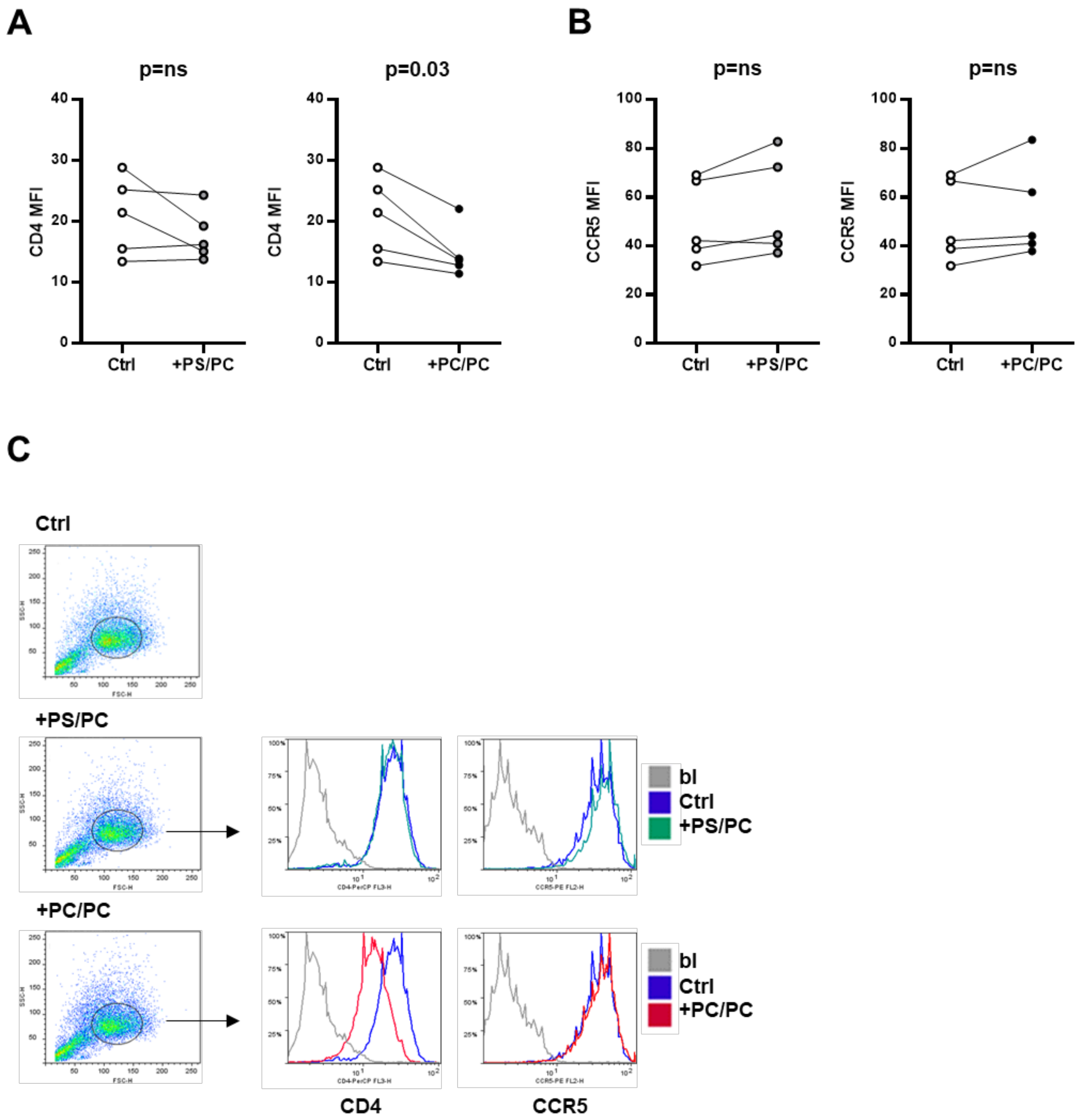

Figure S4. CD4 and CCR5 expression on type-1 macrophages. (A, B) MFI of membrane CD4 **(A**, n=5**)** and membrane CCR5 **(B**, n=5**)** on type-1 macrophage. **(C)** Representative dot plots and overlays. p value was obtained by one-sided Wilcoxon matched-pairs signed rank test (p=ns, not significant).

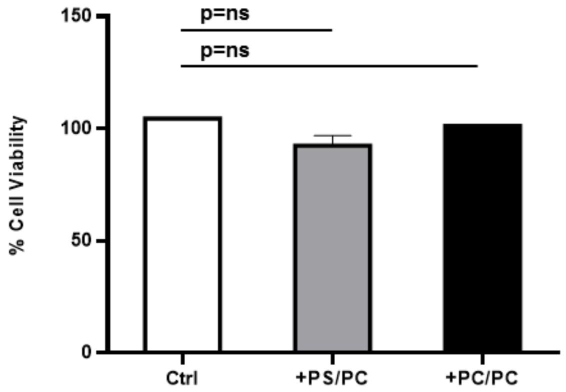

Figure S5. Cell viability of type-1 macrophages following 18 hours of liposome stimulation. Results are shown as means ± Standard Deviation (SD) of % of cell viability of triplicate cultures. % Cell viability = 100 x Experimental OD_{540nm} / Positive Control OD_{540nm}. Data are representative of three independent experiments performed on cells from different healthy donors. p value was obtained by Student's *t* test (p=ns, not significant).

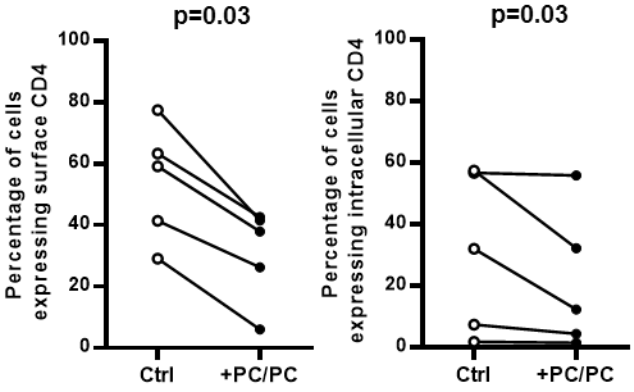

Figure S6. Surface and intracellular CD4 expression on type-1 macrophages following 18 hours of liposome stimulation. Percentage of CD4 positive cells of each healthy donor after 18 hours from PC/PC stimulation (n=5). p value was obtained by one-sided Wilcoxon matched-pairs signed rank test.

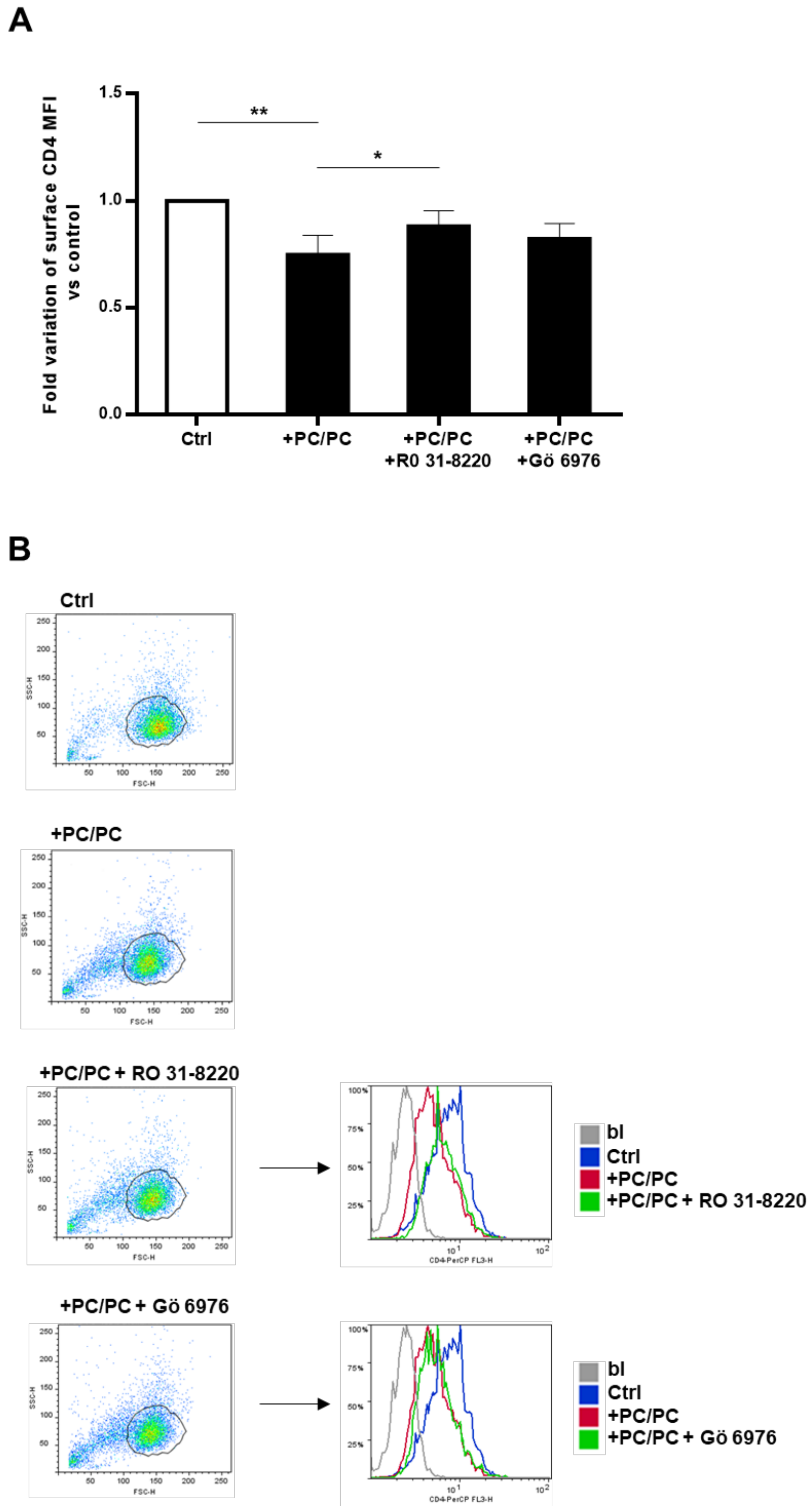

Figure S7. Surface CD4 expression on type-1 macrophages in the presence of PKC inhibitors. (A) Data are shown as mean ± SD of fold variation of CD4 MFI calculated by normalizing the MFI obtained from each healthy donor on their own non-stimulated control (n=5). **(B)** Representative dot plots and overlays. *p<0.05 and **p<0.01 by Student's *t* test. RO 31-8220: PKCs inhibitor; Gö 6976: Ca^{2+}-dependent PKCs inhibitor.

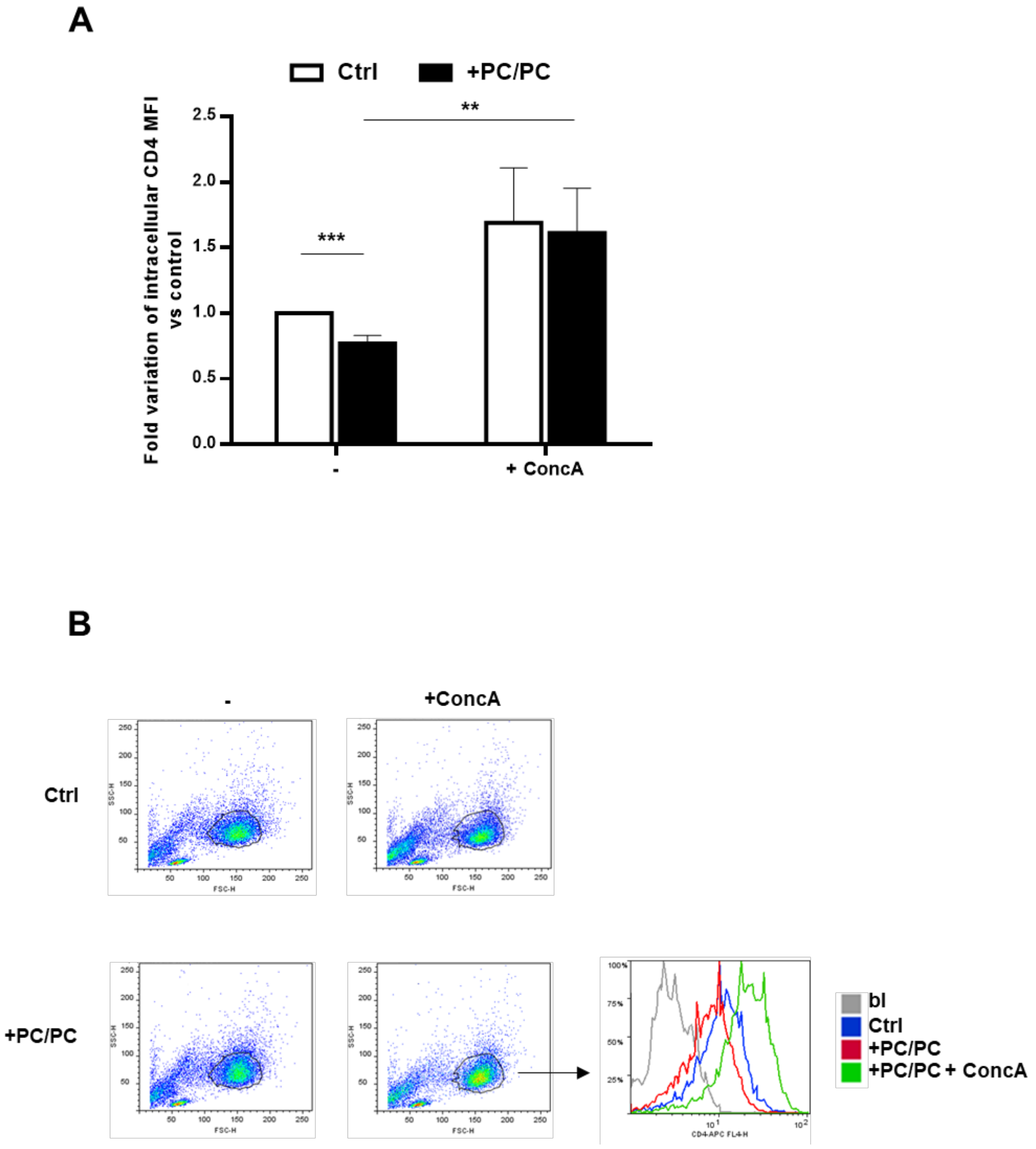

Figure S8. Intracellular CD4 expression on type-1 macrophages in the presence of acidification inhibitor of intracellular compartment. (A) Data are shown as mean ± SD of fold variation of CD4 MFI calculated by normalizing the MFI obtained from each healthy donor on their own non-stimulated control (n=5). **(B)** Representative dot plots and overlays. **$p<0.01$ and ***$p<0.0001$ by Student's *t* test. ConcA: Concanamycin A.
